# Macrophage migration inhibitory factor regulating the expression of VEGF-C through MAPK signal pathways in breast cancer MCF-7 cell

**DOI:** 10.1186/s12957-016-0797-5

**Published:** 2016-02-24

**Authors:** Jinnan Zhang, Guangbo Zhang, Sumei Yang, Junli Qiao, Taixun Li, Song Yang, Yong Hong

**Affiliations:** Guilin Medical University, Guilin, Guangxi Zhuang Autonomous Region 541000 China; First Affiliated Hospital of Soochow University, Suzhou, Jiangsu Province 215000 China; Nanxishan Hospital of Guangxi Zhuang Autonomous Region, Guilin, Guangxi Zhuang Autonomous Region 541000 China

**Keywords:** MIF, VEGF-C, MAPK, Breast cancer

## Abstract

**Background:**

As a kind of versatility of cytokines, overexpression of macrophage migration inhibitory factor (MIF) and vascular endothelial growth factor-C (VEGF-C) have been reported in a wide variety of tumors. However, the correlation and mechanism between MIF and VEGF-C are still not clear. As an important signal transduction system, MAPK signaling pathways participate in a variety of biological behavior of cells. The purposes of this study are to study the relationship between MIF and VEGF-C and discuss the role of MAPK signal pathway in the relationship.

**Methods:**

In this study, we first knocked down the MIF using small interfering RNA (siRNA) and built the stable low expression MIF breast cancer cells (siRNA-MIF-MCF-7) and the negative control cells (siRNA-NC-MCF-7). And then, we evaluated the expression of MIF using Western blot to confirm the effect of transfection. Using real-time fluorescent quantitative polymerase chain reaction and enzyme-linked immunosorbent experiment, we respectively examined the different expression of VEGF-C between siRNA-MIF-MCF-7 and siRNA-NC-MCF-7 and breast cancer cells MCF-7. Moreover, we investigated the expression of p38 MAPK, P-p38 MAPK, p44/42 MAPK, and P-p44/42 MAPK in the three kinds of cells by Western blot to analyze the regulatory mechanism to VEGF-C.

**Results:**

We found that MIF siRNA markedly reduced the expression of MIF. And the expression level of VEGF-C, p38 MAPK, P-p38-MAPK, p44/42-MAPK, and P-p44/42 MAPK in siRNA-MIF-MCF-7 cells had different degree of decrease compared with siRNA-NC-MCF-7 cells and MCF-7 cells.

**Conclusions:**

These results suggest that MIF can regulate the expression of VEGF-C in breast cancer cells. And its regulatory mechanism may work by activating the MAPK signaling pathway.

## Background

Breast cancer is one of the most common malignant tumors in women, and its morbidity is still keeping rising. However, the current treatment is still unsatisfactory. Further research on breast cancer that involved in the development of abnormal factors and its mechanism is of great significance for antitumor therapy of breast cancer. Macrophage migration inhibitory factor (MIF) was first described as a product of activated lymphocytes and named because of inhibiting the random movement of cultured macrophages [1, 2]. As an inflammatory factor, a growing number of studies have shown that MIF is involved in tumor occurrence and development. Overexpression of MIF was reported in numerous tumors such as non-small cell lung cancer [3], gastric cancer [4], breast cancer [5, 6], metastatic melanoma [7], and neuroblastoma [8]. Tumor-associated angiogenesis is of great significance to developing neoplasms. There has been study shown that MIF is overexpressed in breast cancer tissues, cells, and serum of patients. MIF expression level is correlated with the tumor microvessel density, and patients with positive MIF expression show a worse disease-free survival [6]. Through activation of MAP kinase, MIF enhances the vascular endothelial cell growth and differentiation, promotes angiogenesis formation, and leads to support tumorigenesis finally [9, 10], but the mechanism is still unclear.

Vascular endothelial growth factor-C (VEGF-C) is an important family member of the vascular endothelial growth factor. VEGF-C that binds to VEGFR-2 accelerates the growth of vascular and lymphatic; VEGF-C and VEGFR-3, which together have been reported can enhance the permeability of lymphatic vessels, promote proliferation of lymphatic endothelial, and, in addition, induce the lymphangiogenesis and expansion of lymphatic vessel [11]. VEGF-C has been detected in a large variety of malignant human tumors, and the level of expression is associated with lymph node metastasis [12–14]. In particular, the high expression of VEGF-C/VEGFR-3 in invasive breast carcinoma gives rise to lymph node metastasis [15]. VEGF-C plays an indelible role in stimulation the transport of tumor cells by lymphatic vessels and angiogenesis. Although there are a number of studies showing VEGF plays a significant role in promoting the tumor cells growth and metastasis because of the dual function of blood vessels and lymphatic vessels, the mechanism of how the tumor cells stimulate the secretion of VEGF-C is still unclear.

Studies have reported that the expressions of MIF and VEGF are strongly correlated [16]. In a certain range, the exogenous increase of MIF concentration can cause the increase of VEGF expression; furthermore, the production of VEGF can be inhibited by neutralizing MIF mAb [6, 8, 17]. Blocking the MIF receptor (CD74 and CD44) using siRNA can also reduce the secretion of VEGF [17]. Nevertheless, the mechanism of MIF on VEGF is still unclear, and whether it has the same function on VEGF-C so as to influence the formation of tumor lymphatic vessels remains unclear. In the present study, we analyzed the expression of MIF in human breast cancer cell line which was knocked down the MIF messenger RNA (mRNA) using small interfering RNA and expression changes of VEGF-C, p38-MAPK, P-p38MAPK, p44/42-MAPK, and P-p44/42-MAPK to discuss the effect of MIF on VEGF-C and the involved possible molecular mechanisms.

## Methods

### Materials

Human breast cancer cell line (MCF-7) was originally from Shanghai cell bank of the Chinese Academy of Sciences (Shanghai, China). Gene Technology Co., Ltd (Shanghai, China) was entrusted to build Lentiviral vector. Reverse transcription reagent PrimeScript RT Master Mix was purchased from TaKaRa (Kyoto, Japan). Fluorescence quantitative PCR reagent Luminaris Color HiGreen qPCR pre-mixed solution was purchased from Thermo Scientific (Massachusetts, USA). GAPDH and VEGF-C primers were designed and synthesized from Invitrogen (Carlsbad, CA). VEGF-C quantitative enzyme-linked immunosorbent experiment kit was purchased from Siju biological material LTD (Suzhou, China). P38 MAPK, phospho-p38 MAPK, p44/42 MAPK, and phospho-p44/42 MAPK monoclonal antibodies were purchased from Cell Signaling Technology, Inc. (Boston, USA). MIF rabbit monoclonal antibodies were purchased from Abcam trading company LTD (Shanghai, China).

### Cell culture

The experimental cells include the down-regulating expression of MIF cells (siRNA-MIF-MCF-7), negative control cells (siRNA-NC-MCF-7), and blank control cells (MCF-7). The down-regulating expression of MIF cells are the MCF-7 of breast cancer cells which are transfected by lentivirus carrying SiRNA. The negative control cells are the breast cancer cells (MCF-7) which are transfected by lentivirus without SiRNA. And untreated MCF-7 breast cancer cells served as the blank control. All of the cells were cultured in RPMI 1640 containing 10 % heat-inactivated FBS, streptomycin 100 ng/mL, and penicillin 100 U/mL. Cultures were maintained at 37 °C in a humidified incubator in an atmosphere of 95 % air plus 5 % CO_2_.

### Real-time fluorescent quantitative polymerase chain reaction

Total RNA of the three kinds of cells was extracted using TRIzol according to the manufacturer’s protocol and was reverse transcription reagent according to the manufacturer’s instruction. The reaction system of real-time fluorescent quantitative PCR included 10 μL SYBR, 1 μL sense primer, 1 μL antisense primer, 1 μL cDNA, and 7 μL dH2O. The samples were done in triplicate for the target gene and internal control. The PCR primers were designed as follows: VEGF-C, sense primer 5′-TGGCAACATAACAGAGAACAG-3′ and reverse primer 5′-ACCAGGCTGGCAACTTCTAC-3′; GAPDH, sense primer 5′-AGGGGCCATCCATAAACAGTCTTC-3′ and reverse primer 5′-AGAAGGCTGGGGCTCATTG-3′. Quantitative test was carried out 40 cycles under the following conditions: 95 °C for 3 min, 95 °C for 20 s, and 60 °C for 30 s. Calculating the average of each group of samples, the relative transcript level of VEGF-C was analyzed by the formula as follows: ΔCq = target gene Cq − reference gene Cq; ΔΔCq = target gene ΔCq − standard values ΔCq; and the relative copy of target gene = 2^−△△Cq^.

### Enzyme-linked immunosorbent assay

VEGF-C ELISA was performed using a human ELISA kit according to the methods recommended by the manufacturers. Every kind of cells was cultured for 48 h in the medium before the supernatants of cells were collected. The supernatants were centrifuged at 1000 rpm for 10 min to remove the cells and debris and save in freezer keeping in −20 °C.

### Western blot analysis

Cells were seed in six-well plates and washed with PBS before directly lysing in ×1 sample buffer. Proteins were separated by 12 % sodium dodecyl sulfate–polyacrylamide gel electrophoresis and transferred electronically to PVDF membranes. Membranes were blocked with TBST (Tris-buffer saline and 0.2 % Tween-20) containing 5 % non-fat dry milk at room temperature for 1 h and then incubated with anti-MIF (1:4000 dilution) at room temperature for 2 h. The membranes were incubated with the diluted primary antibodies including anti-p38 (1:1000 dilution), anti-P-p38 (1:1000 dilution), anti-p44/42 (1:1000dilution), and anti-P-p44/42(1:2000dilution) at 4 °C overnight. After washed with TBST, they were incubated with anti-goat/rabbit immunoglobulin G-horse radish peroxidase for 1 h and then were washed again. The protein bands were detected whether to be existed by enhanced chemiluminescence (ECL). GAPDH was used as the internal control.

### Statistical analysis

SPSS 13.0 software was used to evaluate the statistical difference of at least three independent experiments. The differences between groups were analyzed by two-tailed Student’s *t* test, with *P* < 0.05 considered significant.

## Results

### Detection VEGF-C expression in the cells by real-time fluorescent quantitative PCR

**Fig. 1:**
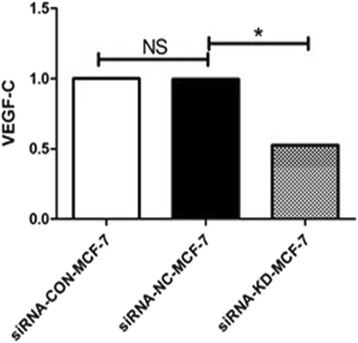
VEGF-C mRNA expression measured by real-time fluorescent quantitative PCR in siRNA-MIF-MCF-7 cells, siRNA-NC-MCF-7 cells, and MCF-7 cells. GAPDH was used as the internal control. The differences between groups were analyzed by two-tailed Student’s *t* test, with *P < 0.05 considered significant. *NS* means there was no statistically significant difference. The experiment was repeated three times independently

The data was analyzed using 2^−△△C^ analysis method. The result showed that the VEGF-C mRNA expression level in siRNA-MIF-MCF-7 cells was remarkable reduced compared with that in siRNA-NC-MCF-7 cells (*P < 0.05). There was no statistically significant difference of the expression of VEGF-C between negative control siRNA-NC-MCF-7 cells and black control MCF-7 cells. Calculated according to the expression of VEGF-C in MCF-7 cells was 100 %, the mRNA expression of VEGF-C inhibition rate in siRNA-MIF-MCF-7 cells was 53 %. Our date showed that VEGF-C expression reduced after reducing MIF expression in breast cancer cells MCF-7. The comparison between different cells is shown in Fig. 1.

### Expression level of VEGF-C in supernatants of cells

**Fig. 2:**
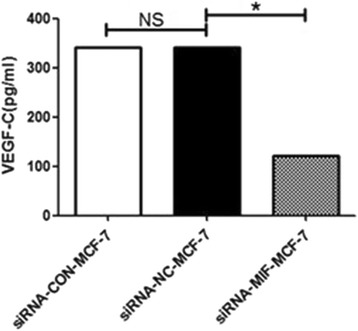
VEGF-C protein level measured by ELISA in the supernatants of siRNA-MIF-MCF-7 cells, siRNA-NC-MCF-7 cells, and MCF-7 cells. The differences between groups were analyzed by two-tailed Student’s *t* test, with *P < 0.05 considered significant. *NS* means there was no statistically significant difference. The experiment was repeated three times independently

Using ELISA, we assessed the VEGF-C secretion in the culture supernatants. In the supernatants of siRNA-MIF-MCF-7 cells, the average concentration was 122 pg/mL. The average concentration of VEGF-C in the supernatants of siRNA-NC-MCF-7 and MCF-7 cultures was 341 and 342 pg/mL, respectively. As shown in Fig. 2, the level of secreted VEGF-C protein in supernatants of the siRNA-MIF-MCF-7 cells was significantly reduced comparing with siRNA-NC-MCF-7 cells and MCF-7 cells (*P < 0.05). Taking VEGF-C expression level of MCF-7 cells as standard, the expression of VEGF-C inhibition rate in siRNA-MIF-MCF-7 cells was 63 %. The difference in supernatants VEGF-C between the siRNA-NC-MCF-7 cells and MCF-7 cells was no statistically significant.

### Expression of MIF, p38-MAPK, P-p38-MAPK, p44/42-MAPK, and P-p44/42-MAPK in cancer cells

**Fig. 3:**
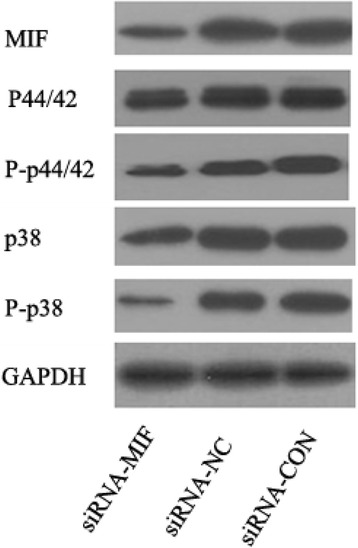
Western blot analysis of MIF, p38-MAPK, P-p38-MAPK, p44/42-MAPK, and P-p44/42-MAPK protein in siRNA-MIF-MCF-7 cells, siRNA-NC-MCF-7 cells, and MCF-7 cells. GAPDH was used as the internal control

Total protein of siRNA-MIF-MCF-7 cells, siRNA-NC-MCF-7 cells, and MCF-7 cells was extracted after 7-day transfection. To define the effects of MIF knockdown, we first detected the expression of MIF using Western blot. Our data showed that MIF siRNA significantly inhibited MIF protein secretion. To further identify MIF signaling pathway, expression and phosphorylation levels of p38 and ERK1/2(p44/42) were detect by Western blot. There results suggested that both ERK1/2 and p38 MAPK signaling pathways were inhibited relative to siRNA-NC-MCF-7 cells and black control MCF-7 cells after knockdown MIF expression (Fig. 3).

## Discussion

MIF secreted by the tumor cells can promote the formation of new blood vessels and regulate the microenvironment of tumor cells, so as to avoid immune surveillance and promote the spread of tumor cells [18, 19]. However, it is still not clear about the way MIF playing a wide range of biological function. In this study, we found that the secretion level of VEGF-C decreased after we knocked down MIF by detecting siRNA-MIF-MCF-7 cells, siRNA-NC-MCF-7 cells, and MCF-7 cells. In addition, the expression and phosphorylation of p38-MAPK and p44/42-MAPK decreased. These results demonstrate that in breast cancer cells, the overexpression of MIF promotes the secretion of VEGF-C, and the MAPK signaling pathway including p38 signaling pathway and ERK1/2 signaling pathway are activated by increasing the phosphorylation level of p38 and ERK1/2 (p44/42) and play a part in biological effects.

VEGF-C combined with VEGFR-3 plays an important role in tumor growth and metastasis. As an important member of the VEGF family, it reportedly induces not only angiogenesis but also lymphangiogenesis. Increased secretion of VEGF-C can promote the lymph node metastasis of a variety of tumors, and it has a more important significance for tumor growth and metastasis [20, 21]. Studies have shown that VEGF-C overexpressed not only in intratumoral regions of breast cancer tissue, its expression in peritumoral regions is higher than that of cancerous tissue, thus promote the formation of lymphatic vessels in the peritumoral areas. This result in the formation of lymphatic vessels is important for tumor growth and metastasis [22, 23]. In human breast cancer cell line MDA-MB-231, IGF-1 can regulate the secretion of VEGF-C by stimulating the MAPK/ERR1/2 signaling pathway, and the application of ERK1/2 inhibitors can block the effect of IGF-1 on VEGF-C secretion [24]. Our study further confirmed the conclusion that it can promote the exocytosis of VEGF-C by activating MAPK signaling pathway. In addition, the study also found that in breast cancer cells, MIF regulates secretion of VEGF-C by influencing stepwise phosphorylation of p38-MAPK and ERK1/2 two classic MAPK signaling pathways. More exact mechanism of the action and their effect of angiogenesis and lymphangiogenesis need further study.

## Conclusions

In conclusion, taking MIF and the related signaling pathway as therapeutic targets is striking. Although there are a lot of research, reports have revealed MIF takes an important role in cancer development, but the exact mechanism is still not clear, as a new tumor therapeutic target is still full of challenges. The results from this study reveal that MIF stimulates the p38-MAPK signaling pathway and ERK1/2 signaling pathway by enhancing the phosphorylation of p38-MAPK and p44/42, meanwhile promote VEGF-C extracellularly export. The discovery provides new train of thought for the treatment of breast cancer.
